# Functional identification of HugZ, a heme oxygenase from *Helicobacter pylori*

**DOI:** 10.1186/1471-2180-8-226

**Published:** 2008-12-17

**Authors:** Ying Guo, Gang Guo, Xuhu Mao, Weijun Zhang, Jie Xiao, Wende Tong, Tao Liu, Bin Xiao, Xiaofei Liu, Youjun Feng, Quanming Zou

**Affiliations:** 1 Department of Clinical Microbiology and Immunology, College of Medical Laboratory, Third Military Medical University, Chongqing 400038, PR China; 2 Department of Blood Transfusion, Chinese People's Liberation Army General Hospital of Military Region, Chengdu, 610083, PR China; 3 The Department of Microbiology, University of Illinois at Urbana-Champaign (UIUC), Illinois, Urbana, 61801, USA

## Abstract

**Background:**

Iron is recognized as an important trace element, essential for most organisms including pathogenic bacteria. HugZ, a protein related to heme iron utilization, is involved in bacterial acquisition of iron from the host. We previously observed that a *hugZ *homologue is correlated with the adaptive colonization of *Helicobacter pylori *(*H. pylori*), a major gastro-enteric pathogen. However, its exact physiological role remains unclear.

**Results:**

A gene homologous to *hugZ*, designated *hp0318*, identified in *H. pylori *ATCC 26695, exhibits 66% similarity to *cj1613c *of *Campylobacter jejuni *NCTC 11168. Soluble 6 × His fused-HugZ protein was expressed *in vitro*. Hemin-agrose affinity analysis indicated that the recombinant HugZ protein can bind to hemin. Absorption spectroscopy at 411 nm further revealed a heme:HugZ binding ratio of 1:1. Enzymatic assays showed that purified recombinant HugZ protein can degrade hemin into biliverdin and carbon monoxide in the presence of either ascorbic acid or NADPH and cytochrome P450 reductase. The biochemical and enzymatic characteristics agreed closely with those of *Campylobacter jejuni *Cj1613c protein, implying that *hp0318 *is a functional member of the HugZ family. A *hugZ *deletion mutant was obtained by homologous recombination. This mutant strain showed poor growth when hemoglobin was provided as the source of iron, partly because of its failure to utilize hemoglobin efficiently. Real-time quantitative PCR also confirmed that the expression of *hugZ *was regulated by iron levels.

**Conclusion:**

These findings provide biochemical and genetic evidence that *hugZ *(*hp0318*) encodes a heme oxygenase involved in iron release/uptake in *H. pylori*.

## Background

*Helicobacter pylori *(*H. pylori*), a Gram-negative microaerophilic spiral bacterium, is known as the major pathogenic agent in a wide range of gastroenteric diseases exemplified by chronic gastritis, peptic ulcer and gastric adeno-carcinoma [1,2]. Increasing evidence suggests that *H. pylori *has adapted particularly to the niche of human stomach. Genetic diversity is widespread among the clinical isolates [3]. This polymorphism can be attributed mainly to the consequence of adaptive changes during colonization, which in turn imply that *H. pylori *has a specialized adaptation mechanism [4-6].

In our earlier study, we harvested several clinical strains of *H. pylori*, which initially grew weakly in Mongolian gerbils but subsequently adapted after 13 serial passages *in vivo *[6]. To elucidate the adaptive colonizing mechanisms of *H. pylori *in Mongolian gerbils further, we applied proteomic approaches to one representative *H. pylori *isolate. Fortunately, four adaptive colonization-associated proteins were identified, among which HugZ (heme iron utilization-related protein) was implicated in adaptive colonization by *H. pylori *for the first time [6]. However, the exact physiological role of HugZ remains elusive.

Iron is regarded as an essential trace element in living organisms, including pathogenic bacteria. It has been suggested that acquisition of iron by *H. pylori *from the host environment is required for colonization, infection and resulting disease [7-9]. Nevertheless, intracellular bacterial iron is precisely regulated and maintained at an appropriate level. Most of the free iron ion in the host is complexed with high-affinity binding proteins such as transferrin in the serum and lactoferrin on mucosal surfaces, so the level of extracellular iron available in the host is extremely low. Consequently, bacterial pathogens including *H. pylori *must have developed some mechanism to compete for the limited host iron for their survival and infection cycle [10-12].

As we know, the siderophore is a common iron acquisition apparatus/system in many pathogens; it obtains iron from transferrin or lactoferrin in the host [10,11]. Other bacteria are also capable of utilizing heme complexes as iron sources. Acquisition can be described as comprising the following steps: binding, uptake and degradation of heme [12]. Some pathogens (such as *Campylobacter jejuni *(*C. jejuni*), *Vibrio cholerae *and *Yersinia entercolitica*) have developed iron-dependent outer membrane receptors specific for heme [13-15]. Heme is transported through such receptors via a TonB-mediated gated pore mechanism [12,15,16], then a periplasmic heme binding protein transports it to the cytoplasmic membrane, where a classical permease/ATPase is thought to transport it actively into the cytoplasm. Once the heme is located within the cytoplasm, a heme oxygenase protein (e.g. hemO) can utilize it. Heme oxygenase is rate-limiting in the degradation process, catalyzing the NADPH-reductase-dependent cleavage of heme to biliverdin with the release of iron and carbon monoxide [17,18]. In *H. pylori*, the mechanism of utilization of heme iron is not yet completely clear. Although several heme iron-repressible outer membrane proteins (IROMPs) might be involved in heme binding and/or uptake [19,20] by *H. pylori*, we still do not know which component functions as the heme oxygenase. In this report, we present the functional identification of HugZ as a heme oxygenase activity in *H. pylori*. Our data imply that the release of iron from heme by HugZ may play a crucial role in the pathogenicity of *H. pylori*.

## Results

### Production and evaluation of homogeneous *H. pylori *HugZ

**Figure 1:**
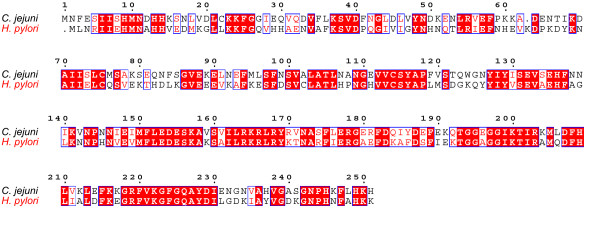
**Amino acid sequence alignment of the *C. jejuni *heme oxygenase (Cj1613c) with *H. pylori *HugZ.** The alignment was performed using the WebESPript 2.2 program on the Institut de Biologie et Chimie des Protéines website.

**Figure 2:**
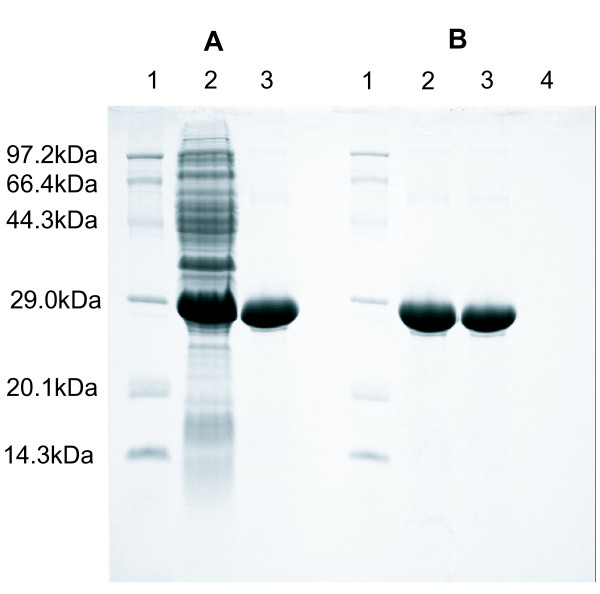
**SDS-PAGE of the purified recombinant HugZ protein and binding of HugZ to hemin-agarose**. (A) *lane 1*, molecular mass markers; *lane 2*, pET22b-*hugZ*/*E. coli *BL21(DE3); *lane 3*, purified 6 × His-HugZ; (B): *lane 1*, molecular mass markers;*lane 2*, purified 6 × His-HugZ;*lane 3*, proteins that bound to the hemin-agarose; *lane 4*, proteins that bound to the hemin-agarose after preincubation with 10 nmol hemin. The data are representative of triplicate independent experiments.

Bioinformatics analysis suggested that a *hugZ *homologue exists in *H. pylori*, which is very similar to that in *C. jejuni *(Fig. 1). To test its activity in iron acquisition, we prepared homogeneous *H. pylori *HugZ protein *in vitro*. Initially, soluble 6 × His-tagged HugZ protein was expressed in a prokaryotic expression system; expression in *Escherichia coli *(*E. coli*) turned the LB medium green (data not shown), implying the presence of a reductase. This observation supports the hypothesis that catalytic turnover of Heme-HugZ triggers the accumulation of biliverdin, which is consistent with the expression profiles of prokaryotic/eukaryotic heme oxygenases [21,22]. The recombinant HugZ protein purified by a Chelating Fastflow XK1610 column (CV = 18 ml) yielded 50 mg/liter and showed about 95% purity on 15% SDS-PAGE (Fig. 2A), indicating high homogeneity. PMF-based sequencing showed that *H. pylori *HugZ is 251 amino acids long and shares 100% similarity to HP0318 (HugZ) protein in ATCC 26695.

**Figure 3:**
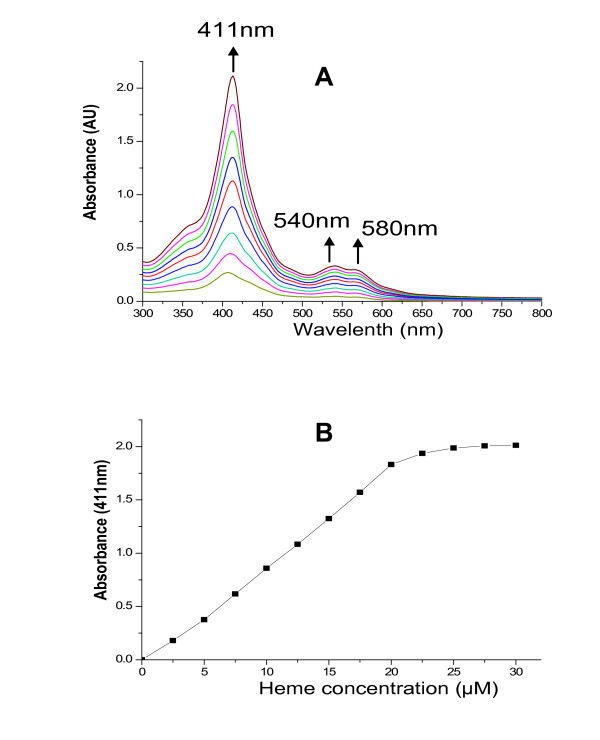
**Absorption spectroscopy of hemin binding to recombinant HugZ**. (A) Absoption profile of hemin at various concentrations. Hemin (2.5 mM in 20 mM NaOH) was titrated in 2.5 μM increments (0, 2.5, 5, 7.5, 10, 12.5, 15, 17.5, 20, 22.5, 25, 27.5, 30 μM) against 20 μM 6 × His-HugZ. Absorbance changes are indicated by the position and direction of the arrows. (B) Hemin: 6 × His-HugZ binding stoichiometry and affinity. Values were plotted as change in absorbance at 411 nm against hemin concentration. The data are representative of triplicate independent spectrophotometric analyses.

To determine whether it is a functional member of the heme oxygenase family, two kinds of heme binding assay were performed. HugZ binding to hemin-agarose beads strongly indicated that it has heme-binding activity (Fig. 2B). Similarly, *in vitro *absorption spectroscopy suggested that HugZ is able to bind heme. As we expected, when HugZ was mixed with hemin, the spectrum of the complex showed a typical spectrographic curve with a prominent Soret peak at 411 nm, and a shoulder at 540 nm and a smaller peak at 580 nm, corresponding to the β- and α-porphyrin bands of the heme-HugZ complex respectively (Fig. 3A). To quantify heme binding, HugZ solution (20 μM) was titrated with increasing amounts of hemin (Fig. 3). The increase in absorption leveled off at approximately 20 μM heme, showing a 1:1 stoichiometry of heme to HugZ (Fig. 3B).

### HugZ catalyzes the degradation of heme

**Figure 4:**
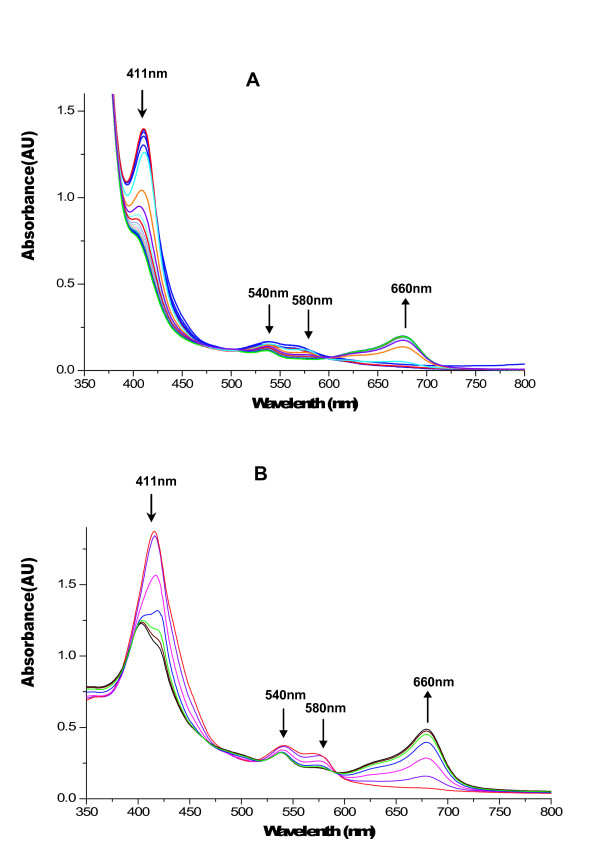
**Degradation of the heme:HugZ complex in the presence of NADPH-cytochrome P450 reductase and ascorbic acid**. (A) Degradation of hemin using recombinant human NADPH-cytochrome P450 reductase as the reductant. Arrows indicate the positions and directions of absorbance change with time. (B) Degradation of hemin using ascorbate as the reductant. The arrow indicates changes in absorption with time. The data are representative of triplicate independent spectrophotometric analyses.

It has been suggested that some heme binding proteins can degrade heme by so-called coupled oxidation, a non-enzymatic mechanism [14]. Coupled oxidation involves the generation of peroxide by the heme protein and is prevented if catalase is present. Heme oxygenases catalyze the opening of the heme macro-cycle in the presence of an electron donor. Purified heme oxygenase has been shown not to release the product biliverdin readily in the absence of biliverdin reductase [21]. Thus, most studies involve single turnover assays, as was done here. In addition, the *in vivo *electron donor for bacterial heme oxygenases is not known, but ascorbate or NADPH-cytochrome P450 reductase may be used for catalysis by the pure enzyme [21,23]. In the first experiment, heme degradation catalyzed by HugZ was measured spectrophotometrically using human NADPH-CPR as the electron donor (Fig. 4A). NADPH was added to the reaction mixture in 10 μM increments and the mixture was scanned from 350 to 800 nm after each addition. The Soret band decreased successively after addition of NADPH. Finally, the HugZ substrate-hemin was exhausted and the NADPH was not oxidized completely, so there was absorption at 340 nm due to NADPH. Heme degradation did not occur if HugZ, NADPH or CPR was omitted from the reaction mixture (not shown).

In the second experiment, the HugZ-dependent disappearance of heme was measured using 20 mM ascorbate as the reductant (Fig. 4B). Heme was degraded more rapidly with ascorbate than with human NADPH-CPR, and most of the decrease was complete by 20 min after the ascorbate was added. No degradation of heme was observed in the absence of HugZ or ascorbate (not shown). Collectively, these findings showed that HugZ catalyzes the enzymatic degradation of heme.

### Biliverdin and CO produced by HugZ-catalyzed heme degradation

**Figure 5:**
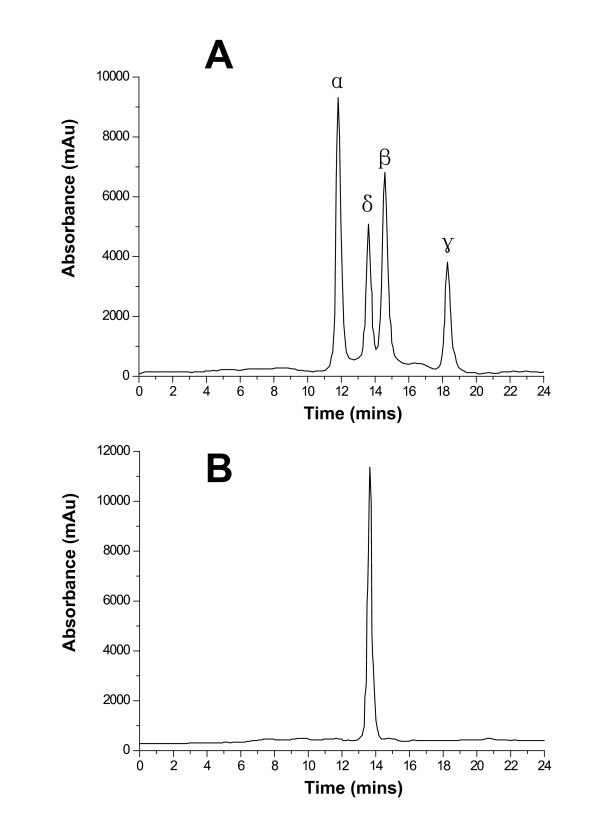
**HPLC detection of the product of the HugZ reaction with NADPH cytochrome P450 reductase**. (A): HPLC chromatogram of a mixture of all four biliverdin isomers as standards. (B): Spectroscopy and HPLC product analysis both show that a product of heme degradation by HugZ is the biliverdin IXδ isomer. The data are representative of three independent HPLC runs.

Biliverdin is the final product of heme degradation by heme oxygenases. When heme was degraded by HugZ, a broad absorbance peak in the 660-nm region became prominent, implying is the presence of biliverdin. To determine the kind of biliverdin formed, we subjected this product to HPLC analysis. HPLC chromatography of all four possible biliverdin isomers is shown for comparison (Fig. 5). The HPLC profiles of the products formed during HugZ-catalyzed heme degradation with ascorbate and NADPH gave a retention time and absorption spectrum identical to that of biliverdin IXδ.

**Figure 6:**
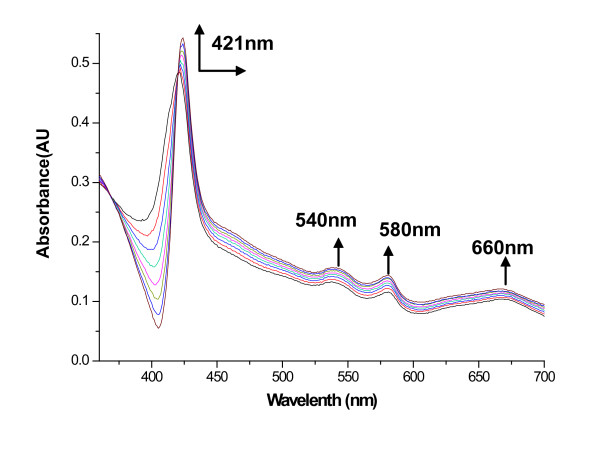
**Difference absorption spectra of the heme-HugZ and NADPH cytochrome P450 reductase reaction in the presence of myoglobin**. The reference and sample cuvettes contained the hemin-HugZ complex (20 μM), recombinant human NADPH reductase (100 μg) and NADPH (100 μM). The reaction was blanked immediately after the addition of NADPH, and myoglobin (125 μM) was added to the sample cuvette. The shift in the Soret band from 411 to 421 nm was monitored at 1-min intervals for 10 min. The data are representative of three independent experiments.

The much higher affinity of myoglobin for CO than for oxygen allows the CO produced by oxidative cleavage of the heme to be detected [21]. Difference absorption spectroscopy in the presence of myoglobin confirmed CO as a product of the oxidative cleavage of heme by HugZ. The myoglobin absorption spectrum was recorded at 2-min intervals in order to monitor the characteristic spectral changes of a myoglobin-CO complex (Fig. 6). The transition of ferrous-dioxygen myoglobin to the ferrous-CO myoglobin complex was associated with a shift in the Soret band from 411 to 421 nm as well as the appearance of bands at 540 and 580 nm. Control reactions in the absence of the heme-HugZ complex showed no shift in the Soret band. The complete conversion indicated that carbon monoxide as well as biliverdin was generated as a product of oxidative heme cleavage in *H. pylori*.

### HugZ is a cytoplasmic protein

**Figure 7:**
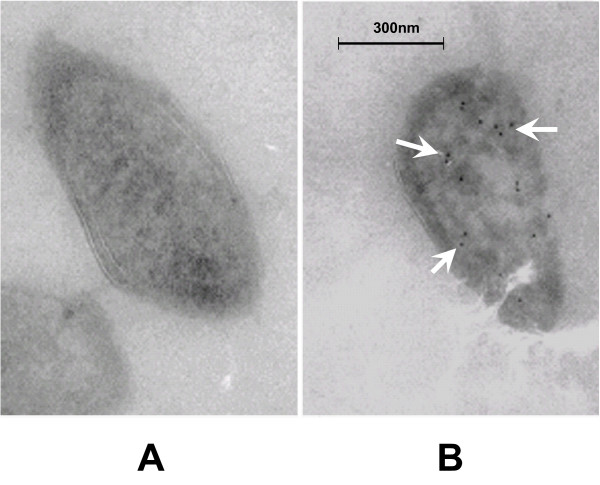
**IEM analysis of HugZ location in *H. pylori *26695**. Representative immuno-electron micrographs of frozen thin-sectioned specimens are shown. Labeling in the *H. pylori *cytoplasm (B) was strong in comparison to the negative control (A). The cell wall shows different widths depending on the plane of section. Scale bar, 300 nm.

To determine the cellular location of HugZ, Immunoelectron microscopy (IEM) was performed. Frozen sectioned samples of *H. pylori *26695 strains were treated with anti-HugZ antibodies and gold-labeled secondary antibodies. Analysis of the positions of the gold particles (Fig. 7B) revealed that HugZ was predominantly located in the cytoplasm in *H. pylori *cells.

### The *hugZ *mutant fails to utilize heme iron for normal growth

**Figure 8:**
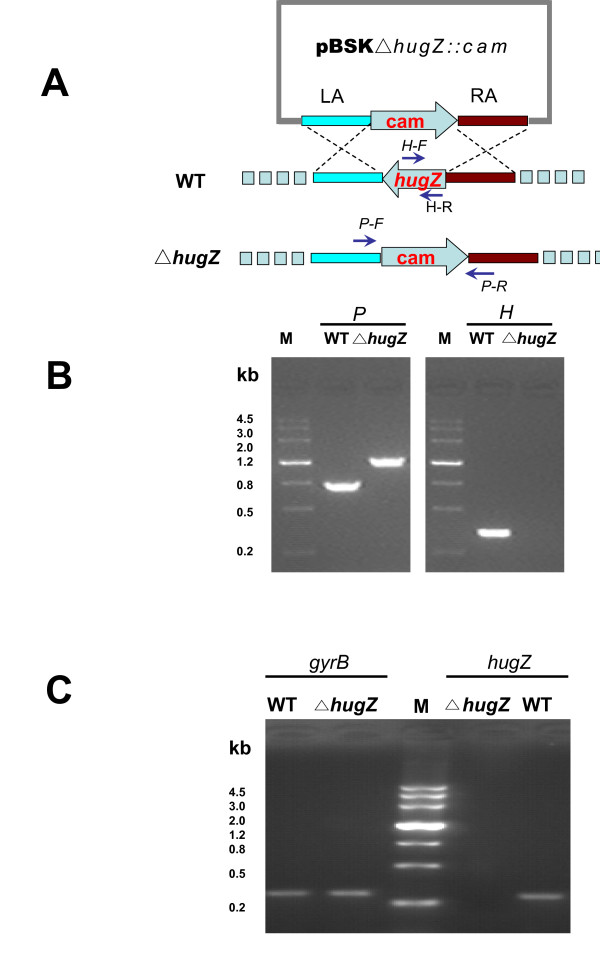
**Identification of *hugZ*, the isogenic mutant of *hugZ *in *H. pylori *26695**. (A) Cartoon description of *hugZ *knockout from the *H. pylori *26695 chromosome. pBSKΔ*hugZ*::*cam *is the recombinant vector constructed specifically to inactivate *hugZ*. *hugZLA *and *hugZRA *respectively indicate the left- and right- border of *hugZ*. A pair of specific primers (*P-F *&*P-R*) located adjacent to *hugZ *on both sides are indicated with blue arrows and used for PCR-detection of *hugZ *in the *H. pylori *26695 genome. WT, wild type *H. pylori *26695; Δ*hugZ*, an isogenic mutant of gene *hugZ*. (B) Multiple-PCR analysis of Δ*hugZ*. The PCR products were separated by electrophoresis on a 1.0% agarose gel stained with ethidium bromide (EB). P & H, the PCR product amplified with the *P-F *&*P-R *and *H-F &H-R *primers for *hugZ*. Δ*hugZ *has been replaced by *Cam*^*R *^without affecting either boundary sequence (not shown). (C) RT-PCR analysis of Δ*hugZ *using *hugZ1 & hugZ2 *and *gyrB1 & gyrB2 *primers. RT-PCR products of *hugZ *and *gyrB *were separated by electrophoresis on a 2.0% agarose gel.

In order to elucidate the role of HugZ, the mutant Δ*hugZ *was obtained from more than 100 *H. pylori *transformants. The correct genotype of Δ*hugZ *was systemically confirmed by PCR (Fig. 8A & 8B), RT-PCR (Fig. 8C) and direct DNA sequencing (data not shown).

**Figure 9:**
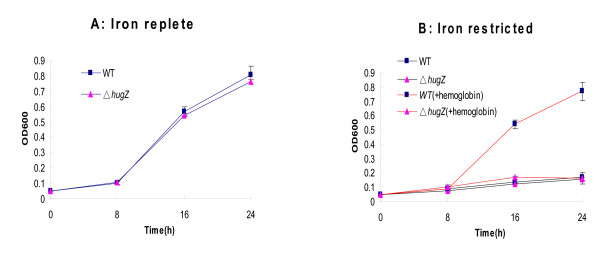
**Growth of *hugZ *mutants**. Samples were tested in triplicate, and the data plotted are the means of three independent experiments together with the sample error. Symbols: A: *H. pylori *26695 WT and Δ*hugZ *strains grown in BBF supplemented with 50 μM FeCl_3 _(iron-replete); B: WT andΔ*hugZ *strains grown in BBF plus 75 μM desferal (iron-restricted) with or without 12.5 μM Hb supplement. The optical density of the bacteria was monitored at 600 nm. The key to symbols is shown in figure. Error bars indicate standard deviation from three replicate cultures. Hb, hemoglobin.

The *hugZ *deletion mutant (Δ*hugZ*) grew normally in liquid BBF and on BBF blood agar plates, indicating that HugZ is not required for bacterial growth under iron-replete conditions. Subsequently, we tested its growth in the presence of different iron sources. Δ*hugZ *strains showed poor growth in iron-restricted conditions while the wild type grew well (Fig. 9). These data suggest that the *hugZ *mutant cannot utilize heme iron for normal growth.

### Regulation of *hugZ *expression by iron

**Figure 10:**
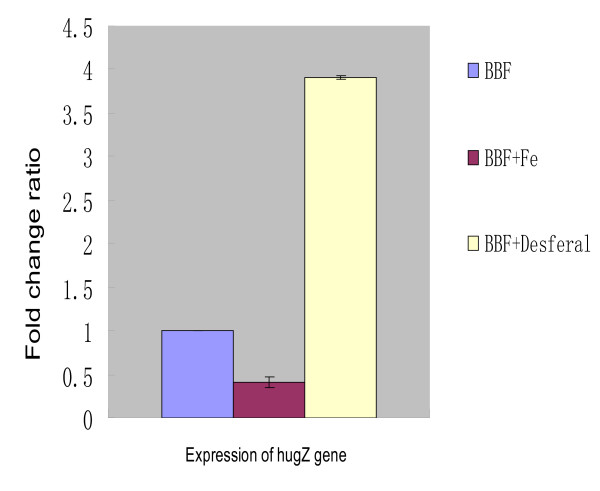
**Comparison of the levels of *hugZ *expression under different iron level conditions, detected by real-time quantitative RT-PCR**. The results are based on the ratio *hugZ *mRNA amplification/*gyrB *mRNA amplification, which are presented as the fold induction of mRNA expression relative to the amount present in BBF. Real-time PCR was conducted in duplicate for each sample and the mean value was calculated. Expression values were calculated from three biological replicates. (BBF: Brucella Broth with 10% fetal bovine serum; BBF+Fe: BBF plus 50 μM FeCl_3; _BBF+Desferal: BBF plus 75 μM Desferal).

Merrell *et al*. reported that *hugZ *(*hp0318*) was one of the genes induced by iron starvation [24]. To test whether *hugZ *is regulated by iron, real-time quantitative PCR was performed. The effects of different iron levels on *hugZ *transcription varied (Fig. 10). Transcription was suppressed by FeCl_3 _(compared to BBF, the change fold ratio was 0.410 ± 0.056 (*p *< 0.01, Student's *t*-test)) and stimulated under iron-restricted conditions (compared to BBF, the change fold ratio was 3.90 ± 0.010 (*p *< 0.01, Student's *t*-test)). These results indicated that *hugZ *(*hp0318*) is down-regulated by iron.

## Discussion

A wide array of metal ions including iron, copper and nickel are known to be closely related to *H. pylori *colonization and infection [25,26]. Iron metabolism-related proteins play important roles in *H. pylori *infections. However, the iron-specific metabolic mechanism in *H. pylori *is still not well understood. Bacteria require iron to complete their life cycles and in particular for growth and infection. The limited availability of extra-cellular iron in the host, which is partly due to iron insolubility, restricts microbial growth greatly, so iron acquisition seems to be crucial for the survival of pathogens. Actually, it has been suggested that bacteria evolve sophisticated systems to compete for iron with their hosts. In general, heme is an important iron source in hosts and it can be utilized by most pathogens. Heme is degraded by heme oxygenase in the bacterial cytoplasm, releasing the iron.

Heme oxygenase is the rate-limiting enzyme in heme degradation; it catalyzes reduction system-dependent cleavage of heme to biliverdin with the release of iron and carbon monoxide. It is reasonable to suppose that bacterial heme oxygenase releases the iron from heme for subsequent use by the invading pathogen [18]. Heme oxygenases are widespread among pathogenic bacteria such as *C. jejuni *and *Y. pestis *and play key roles in the growth and colonization of those pathogens [13,27]. Heme oxygenase mutants of *Corynebacterium diphtheriae *and *Neisseria meningitidis *were unable to utilize heme or hemoglobin as an iron source [28,29]. Similarly, it has been suggested that heme oxygenase (Cj1613c) is necessary for growth in *C. jejuni *[13]. For *H. pylori*, the role of heme degradation in iron metabolism is relatively obscure. In this study, we identified a heme oxygenase called HugZ that is responsible for heme iron utilization in *H. pylori*. The heme oxygenase activity of HugZ was confirmed by the appearance of characteristic spectral changes following addition of ascorbic acid or a NADPH-CPR system as electron donor. HugZ binds to hemin *in vitro *at 1:1 and produces absorbance bands at 411, 540 and 580 nm, which are similar to those reported for other heme oxygenases such as ChuS [30] and Cj1613c [13]. The formation of a broad absorbance band at 395 and 660 nm suggests that the end product of heme degradation is iron-free biliverdin rather than ferric biliverdin [28].

We demonstrated that the products of *hugZ *cleave heme to carbon monoxide and biliverdin IXδ, which shows that the δ-meso carbon bridge position in the heme precursor is eliminated by HugZ. As with various eukaryotic and prokaryotic heme oxygenases, overexpression of HugZ in *E. coli *can make the culture medium green owing to the accumulation of biliverdin. It is presumed that during the expression of those exogenous heme oxygenases in *E. coli*, reducing systems in the bacterium support the catalytic turnover of heme [21,22]. Furthermore, several reduction systems including human CPR and ascorbic acid can support biliverdin production by purified recombinant HugZ *in vitro*. These results suggest that *H. pylori *probably has the same reduction partners for HugZ-heme oxygenase activity.

Further experiments showed that the *H. pylori hugZ *mutant exhibited poor growth, though wild type strains grew well when heme was added to iron-restricted BBF, indicating that the *hugZ *mutant cannot effectively utilize hemoglobin as a heme iron source, which confirmed that HugZ is a heme oxygenase.

In mammals, the primary function of heme oxygenases is to maintain iron homeostasis, whereas prokaryotic heme oxygenases help bacteria to take in iron from heme. Most bacterial heme oxygenases are regulated by the ferric uptake repressor (Fur). Fur requires iron to bind to target DNA sequences (Fur-boxes) and controls the expression of iron-regulated genes [18]. Merrell and Gancz *et al*. used a Microarray to analyze the expression of iron-regulated genes in *H. pylori *and reported that *hp0318 *(*hugZ*) is one of the genes induced by iron starvation; they presumed that a hypothetical Fur box located before *hp0321 *controls *hp0318 *expression [24,31]. Our real-time quantitative RT PCR results also support that the view that *hugZ *(*hp0318*) is down-regulated by iron. However, further studies are needed to determine whether the presumed Fur box controls the transcription of *hugZ*.

## Conclusion

Taken together, these findings confirm that *H. pylori *HP0318 (HugZ) is a heme oxygenase. Our data imply that HugZ may play a crucial role in the acquisition of heme iron by *H. pylori*.

## Methods

### Bacterial strains and growth conditions

*H. pylori *strain ATCC 26695 was cultivated in liquid Brucella Broth with 10% fetal bovine serum (BBF) and a mixture of antibiotics (10 μg/ml vancomycin, 5 μg/ml trimethoprim, 6 μg/ml nalidixic acid and 5 μg/ml amphotericin B). The solid medium consisted of the aforementioned ingredients with 5% rabbit blood and 1.5% agar at 37°C under microaerobic conditions (10% CO_2_, 85% N_2_, 5% O_2_) [32]. Iron-replete conditions were achieved by adding FeCl_3 _to a final concentration of 50 μM. Iron-restricted conditions were achieved by adding the iron chelator desferrioxamine mesylate (Desferal) to a final concentration of 75 μM [19]. *H. pylori *strains were grown in the presence of heme as the sole iron source at a final concentration of 12.5 μM hemoglobin in iron-restricted BBF. The strains were initially cultured on BBF blood agar plates overnight, harvested in a suitable volume of BBF, and used to inoculate 5 ml BBF to an optical density at 600 nm (OD_600_) of 0.05. The cultures were incubated microaerobically with shaking, and the optical density was monitored at regular time intervals.*E. coli *strains DH5α and BL21 (DE3) were used as cloning host and expression host, respectively. Antibiotic selection was achieved when necessary by addition of ampicillin (100 μg/ml) or chloramphenicol (10 μg/ml).

### Construction of *hugZ *knockout mutant

*hugZ *was activated by allelic replacement with a constitutively-expressed chloramphenicol resistance (Cam^R^) cassette. First, the DNA sequences flanking hugZ, including 1000 base pairs upstream and 1000 base pairs downstream, were amplified from the chromosomal DNA of *H. pylori 26695 *using PCR with two pairs of specific primers (*LA-F1/LA-R1 *and *RA-F1/RA-R1*) carrying *SacI*/*XbaI *and *SmaI/SalI *restriction enzyme sites, respectively (Table 1). After digestion with the corresponding restriction enzymes, the DNA fragments were cloned directionally into a pBluescript II SK(-) vector. The Cam^R ^gene cassette (from phel2 [33]) was then inserted at the XbaI/SmaI sites to generate the *hugZ *knockout vector pBSKΔ*hugZ::cam*^*R*^. To obtain the isogenic mutant Δ*hugZ*, the plasmid pBSKΔ*hugZ::cam*^*R *^was electrotransformed into *H. pylori *26695, where the Cam^R ^marked mutation was introduced into the genome by homologous recombination, resulting in the *hugZ::cam*^*R *^mutant strain. PCR was used to examine all the Cam^R ^transformants with a series of specific primers.

**Table 1:** Oligonucleotide primers used in this study

**Primer**	**Sequence(5' → 3')**	**Characteristics**	**Functions (genes)**
*H1*	CGCGCATATGCTTAATCGTATC	*NdeI*	To amplify gene *hugZ*
*H2*	GGCCTCGAGTTTCTTGTGAGCG	*XhoI*	
*LA-F1*	CGAGCTCCCATTACTACTGCTACTACTA	*SacI*	To amplify left arm of gene *hugZ *(LA)
*LA-R1*	GCTCTAGAGCACCGCTCATAAGGGGCAA	*XbaI*	
*RA-F1*	TCCCCCGGGGGAAATATTCTCCTTAGTT	*SmaI*	To amplify right arm of gene *hugZ *(RA)
*RA-R1*	ACGTCGACGCAATGCTTTTAGAAATTAA	*SalI*	
*H-F*	TCGCTAAACAAACAGAATCAA	/	To detect *hugZ*
*H-R*	ATGTGTTCTATGATACGATTAAGCAT	/	
*P-F*	GCTCTAGAGCTTGCCCCTTATGAGCGGT	/	To detect *hugZ *or *cam*
*P-R*	CTTATTTTTTGAAACTAAGGAGAATATT	/	
*hugZ-1*	TTGGGCAAGTCCATCACG	245 bp	RT-PCR/Real-time RT-PCR Evaluation of *hugZ*
*hugZ-2*	GGTCGCTAAACAAACAGAA		
*gyrB-1*	CGCTAAAGAAAGTGGCACGA	267 bp	RT-PCR/Real-time RT-PCR Evaluation of *gyrB *(normalizer)
*gyrB-2*	TGCGCGTTTCTTCATCCAT		

The underlined sequences are the restriction sites. /: Absence of restriction endoenzyme sites.

### Overexpression and purification of recombinant HugZ and preparation of HugZ antiserum

After the amino acid sequence of HugZ (HP0318 located in *Helicobacter pylori *26695) was aligned with that of Cj1613c of *C. jejuni *NCTC 11168 [13,32], it was recognized as a candidate/functional member related to iron acquisition. To test this bioinformatics-based hypothesis, the full length 753 bp *hugZ *was amplified with primers *H*_1 _(with *NdeI *site) and *H*_2 _(with *XhoI *site) (Table 1), using genomic *H. pylori *ATCC 26695 DNA as template. The PCR conditions were initial denaturation for 10 min (95°C) followed by 35 cycles of amplification (40 s at 95°C, 30 s at 53°C and 1 min at 72°C) and a final extension for 10 min at 72°C, using a gene cycler (BIORAD). The PCR product was cloned into the pMD-18T vector (Takara), generating pMD-18T-*hugZ*, and directly subcloned into pET-22b (+) (Novagen) via *Nde *I and *Xho *I restriction sites, resulting in the recombinant expression plasmid pET-22b-*hugZ*. Finally, the positive clones were further confirmed by direct DNA sequencing. For the expression of HugZ protein, an overnight culture of strain BL21 (pET22b-*hugZ*) was diluted 1:100 into 2000 ml of LB medium; 0.5 mM IPTG (isopropyl β-D-thio β-D-galacto-pyranoside; Sigma) was added when the OD_600 _reached 0.6 and the culture was maintained for 12 h at 16°C with shaking. Cells were harvested by centrifugation and resuspended in 200 ml of 20 mM Tris-HCl 0.5 M NaCl (pH 7.8). After homogenization 5 times in an APV1000 High Pressure Homogenizer (Denmark) at 750 bar on ice, the sample was centrifuged at 12,500 × g for 30 min and filtered through a 0.45- μm-pore-size filter (Sartorius). The recombinant HugZ was purified using the AKTÄ Explorer100 system with a Chelating Fastflow XK1620 column (CV = 18 ml) (GE) in accordance with the manufacturer's standard protocol. Protein purity was determined by SDS-PAGE. Also, Peptide Mass Fingerprint (PMF) analysis was used to identify the HugZ protein (Beijing Genomics Institute). The purified HugZ protein was concentrated and dialyzed three times (Vivaspin 20 centrifugal concentrators, 10 kDa molecular weight cut off, Sartorius) against 20 mM Tris-HCl (pH 7.8) at 4°C and quantified by the Lowry Method (600 μg/ml). The purified protein was used to prepare anti-HugZ antiserum in rabbits in accordance with standard protocols.

### Immunoelectron microscopy (IEM)

IEM was performed as described by Michie *et al*. [34]. Wild type *H. pylori *26695 cells were grown in BBF at 37°C overnight, fixed with 10% glutaraldehyde and washed before dehydration at 4°C in 80% ethanol. The cells were frozen in liquid nitrogen for use. For immunolabeling, frozen ultrathin sections were collected on Formvar-coated gold slot grids. Sections were treated with blocking buffer then incubated for 4 h at room temperature with affinity-purified anti-HugZ rabbit antiserum diluted 1:2000 in BB or with BB alone (as a negative control). The grids were washed six times with wash buffer (PBS 0.05% Tween 20), blocked with 5% normal goat serum and incubated in goat anti-rabbit 15-nm gold diluted 1:100 in BB plus 5% goat serum. The grids were washed twice with wash buffer, twice with PBS and twice with water before staining in saturated aqueous uranyl acetate for 20 min. Sections were viewed under a Philips Tecnai 10 transmission electron microscope.

### Binding of HugZ to hemin

Two independent assays, which involved hemin-agarose beads and spectrophotometry, were utilized to test the binding activity of HugZ. The hemin agarose-based assay was performed as described by Lee [35]. In brief, 100 μl of hemin-agarose (Sigma-Aldrich) was washed thrice in 10 ml 0.5 M NaCl-20 mM Tris-HCl (pH 7.8), then incubated with purified HugZ (20 μg) with or without 10 nmol hemin for 30 min. After the removal of contaminants, the bound protein was analyzed by 15% SDS-PAGE. The absorption spectroscopy assay was carried out as described by Wilks *et al*. [21]. One milliliter of 20 μM HugZ (in 20 mM Tris-HCl (pH 7.8)) was applied at 25°C. Hemin (2.5 mM in 20 mM NaOH) was titrated against the HugZ in 2.5 μM increments and the absorbance spectrum between 300 and 800 nm was recorded on a TU-1901 spectrophotometer (Pgeneral, China). The absorption at 411 nm was plotted against the heme concentration.

### Determination of hugZ heme oxygenase activity

Heme-HugZ complex was prepared at a hemin:protein ratio of 2:1 and excess heme was removed by filtration through a Sephadex G-25 column. Degradation of HugZ-bound hemin to biliverdin was mediated by two electron-donor systems (ascorbic acid and NADPH-CPR). Ascorbic acid was added to a final concentration of 20 mM. In the NADPH-CPR system, heme-HugZ protein (20 μM) was added to 100 μg of recombinant human NADPH-cytochrome P450 reductase (CPR). The reaction was initiated by adding NADPH to 100 μM and spectra were recorded from 350 nm to 800 nm every 2 min for 1 h. In order to avoid the involvement of non-enzymatic H_2_O_2_-mediated conversion of heme to biliverdin, 2 μM catalase (bovine liver, Sigma-Aldrich) was added to the reaction systems [13]. Finally, two heme-HugZ reaction products, biliverdin and carbon monoxide (CO), were determined. First, HPLC was used to detect biliverdin [21]. Second, to determine CO, recombinant human NADPH-CPR (100 μg) and NADPH (100 μM) were placed in both the reference and reaction cuvettes in a final volume of 3 ml and blanked immediately. Then 150 μl of myoglobin (125 μM) (Sigma-Aldrich) was added to the reaction cuvette and the same volume of buffer to the reference cuvette. Spectra were recorded every 2 min between 400 and 700 nm for up to 1 h [13].

### Transcriptional analysis of *hugZ *by real-time RT-PCR

*H. pylori *RNA was isolated using TRIzol reagent (Gibco/BRL). The RNA concentration was quantified by the OD_260_, and RNA integrity was verified by visualization on a 2% agarose gel. Real-time quantitative PCR was performed as described by Feng *et al*. with a minor modification [36]. Briefly, *hugZ*-specific primers (*hugZ1 *and *hugZ2*) (Table 1) and SYBR Green PCR master mix (ABI) were used. Real-time PCR was performed using a Rotor-Gene 6000 real-time PCR system (Corbett Life Science, Australia). Known concentrations of *H. pylori *26695 genomic DNA were used to construct a gene-specific standard curve so that the concentration of template in each reaction could be determined. The gene encoding DNA gyrase subunit B, *GyrB *(*HP0501*) [37], was used to normalize all reactions. Melting curve analysis confirmed that all PCRs amplified a single product.

## Authors' contributions

QZ, GG and XM conceived and designed the experiments. YG, GG, WZ, JX, TL, BX and XL performed the experiments. YG, YF, GG and XM analyzed the data. GG, WT and YF contributed reagents/materials/analysis tools. YG, YF and GG wrote the paper.
